# Comparison of monitor unit calculations performed with a 3D computerized planning system and independent “hand” calculations: Results of three years clinical experience

**DOI:** 10.1120/jacmp.v3i4.2553

**Published:** 2002-09-01

**Authors:** Jackson Chan, David Russell, Victor G. Peters, Thomas J. Farrell

**Affiliations:** ^1^ Department of Medical Physics Hamilton Regional Cancer Centre 699 Concession St. Hamilton Ontario Canada L8V 5C2

**Keywords:** Photon dosimetry, monitor unit calculations, x‐ray dose algorithm verification

## Abstract

A comparison of the monitor unit calculations of a commercial 3D computerized treatment planning system (TPS) with “hand” calculations from lookup tables was made for a large number of clinical cases (greater than 13 500 treatment fields). Differences were analyzed by treatment site for prostate, rectum, cranium, and breast. The 3D TPS monitor unit calculation was systematically higher than the “hand” calculation by an amount that depended on the complexity of the treatment geometry. For simple geometries the mean difference was 1% and was as high as 3% for more complicated geometries. The higher value was attributed to an accumulation of differences introduced by multiple factors in the monitor unit calculation. Careful attention to factors such as patient contour could reduce the mean difference. “Hand” calculations were shown to be an accurate and useful tool for verification of TPS monitor unit calculations.

PACS number(s): 87.53.‐j, 87.52.‐g

## INTRODUCTION

Commissioning of a radiation treatment planning system (TPS) should include extensive tests to confirm the validity of monitor unit calculations.^1^ ^,^ ^2^ Several studies^3^ ^,^ ^4^ ^,^ ^5^ have been published showing that an accuracy of 1–2% can be achieved along the central axis of open or wedged beams in standard “commissioning” geometry, i.e., normal incidence to a flat phantom usually composed of water. Errors increase for more complex geometries. Although the validity of the calculation algorithms can be tested during the commissioning of a TPS, good clinical practice further requires that all monitor units calculated for clinical use be verified using a second independent calculation method. Although modern planning systems use sophisticated algorithms for dose calculation, verification of the monitor units calculated by the TPS is typically performed using a “hand” calculation based on look‐up tables of standard beam data. In principle, “hand” calculations are expected to be less accurate than those performed by the TPS because factors such as patient surface convexity or beam obliquity are not considered. However, the significance of observed differences may be difficult to determine. Starkscall *et al.* ^6^ have shown that a comparison of monitor unit calculations is useful as a means of identifying systematic errors in the dose calculation algorithm or in the implementation of the algorithm by the TPS. Leszczynski and Dunscombe^7^ concluded that for the Helax‐TMS planning system (Helax AB, Uppsala, Sweden) it is possible in typical clinical situations to corroborate the dose calculation to a reference point using a standard manual calculation method. In this paper we present a comparison of the monitor unit calculations of our planning system, Pinnacle^3^ (ADAC Laboratories, Milpitas, CA), with “hand” calculations for a large number of clinical cases. The purpose was to evaluate the accuracy and utility of the “hand” calculation as a verification tool in several treatment sites covering a range of treatment complexity.

**Table I:** Factors used for “hand” monitor unit calculation.

Factor	Symbol	Definition (dependence)
Output calibration	*K*	Dose in cGy/MU in calibration conditions. 1 cGy/MU at SAD for reference depth and field size.
Collimator scatter factor	$S_{c}$	Dose rate in air for a given collimator setting relative to that for the reference collimator setting (field size).
Phantom scatter factor	$S_{p}$	Dose rate at reference depth for a given field relative to that at the same depth for the reference field, using the same collimator setting (depth, field size).
Tissue phantom ratio	TPR	Dose rate at depth relative to dose rate at the reference depth for the same field size (depth, field size).
Tray attenuation factor	TF	Attenuation factor due to shielding tray (none).
Wedge attenuation factor	WF	Attenuation due to transmission through physical wedge (depth, field size).
Off‐axis ratio	OAR	Dose rate at off‐axis position relative to dose rate at the central axis (off‐axis distance).
Wedge off‐axis ratio	WOAR	Attenuation through wedge at off‐axis position relative to that through the central axis (off‐axis distance).
Inverse square correction	ISC	Dose rate in air at prescription distance relative to that at standard SAD (depth+SSD).

## MATERIALS AND METHODS

The monitor units calculated using our treatment planning system, Pinnacle (ADAC Laboratories, Milpitas, CA),^3^ are used for treatment delivery. Dose is prescribed to a single reference point inside the planning target volume as described by (ICRU) reports.^8^ ^,^ ^9^ Monitor units are calculated using a direct forward calculation of energy fluence using the convolution‐superposition algorithm and the machine model parameters which best fit our commissioning data. Details of the method are described elsewhere,^10^ ^,^ ^11^ but in general the commissioning procedure consists of matching measured percent depth dose and beam profiles by adjusting model parameters.

The accuracy of the monitor unit calculation was assessed during commissioning by comparison with measured dose rates for a variety of open and wedged beams in a water phantom. In general the accuracy is similar to results reported by other investigators.^12^ For 6 MV, except for highly elongated rectangular fields, the differences were better than ±1% and often better than ±0.5% at typical treatment depths. Errors could be as large as 2% for more irregular fields. For 18 MV errors were usually less than ±1% at typical treatment depths but could be as high as ±2% for some fields. Cobalt errors were similar. All these errors were random and there is no evidence of any systematic differences between the measured and the TPS calculated monitor units.

The monitor units are verified using an in‐house computer program. This program is a computerized “hand” calculation that follows the formalism described by Khan.^13^ A general equation describing this calculation is as follows:

(1) $$MU=\frac{Prescribed\;dose}{K●S_{c}●S_{p}●TPR●TF●WF●OAR●WOAR●ISC}.$$

The factors in this equation are defined in Table I and the details of how they are applied will be discussed below with respect to individual treatment sites.

Values for most of these factors are obtained from look‐up tables based on direct measurements using an ionization chamber in a water phantom. These data are an independent data set from the percent depth dose data used to commission the TPS. The factors TPR, TF, WF, OAR, and WOAR were all measured for a grid of different field sizes and depths. If a factor was insensitive to either field size or depth (accuracy better than 1%) the look‐up table for that factor was collapsed to an average value over the measured range. The dose rate under calibration conditions, *K*, is set to be the same for both “hand” calculations and TPS calculations. The calibration depth was chosen to be greater than the range of contamination electrons and is the same for hand and TPS calculations for 6 and 18 MV photons. The calibration distance is at 100 cm.

**Table II:** Statistical summary of MU ratios by treatment site.

Statistic/Site	Prostate	Rectum	Brain	Breast
Number of fields	3577	1948	741	7510
Average MU ratio	1.010	1.011	1.013	1.012
St. dev. MU ratio	0.005	0.008	0.008	0.016
Min. MU ratio	0.992	0.984	0.977	0.954
Max. MU ratio	1.034	1.051	1.043	1.077

The “hand” calculations are used to verify the TPS calculations, and the ratio of the TPS monitor unit calculation to the “hand” calculation is generated for each treatment field,

(2) $${MU}_{ratio}=\frac{{MU}_{TPS}}{{MU}_{HC}}.$$

MU ratios are recorded for every treatment field calculation in a master data base. We analyzed these values for four common treatment sites representing different levels of complexity over a three‐and‐one‐half‐year period from May 1998 to December 2001. Statistics describing the distributions of MU ratios were generated and analyzed. A random subset of 100 patients charts were reanalyzed in order to verify the accuracy of the MU ratios in the patient database. There were no transcription errors found. In addition all data for any suspected outlier (differences of greater than 5%) was verified from patient charts.

Tissue heterogeneities were not considered for either the TPS monitor unit calculation or the “hand” calculation. The four treatment sites are described below in order of increasing calculation complexity.

Prostate: Dose was prescribed to the isocenter using a four‐field box technique and 18 MV x rays. All patient contour data was acquired from computed tomography (CT) data. The patient contour was a simple, nearly flat surface for each treatment beam. Shielding usually consisted of small corner blocks encompassing less than 15% of the treatment field. The “hand” calculation consisted of table look‐up of the tissue phantom ratio (TPR) as a function of field size and depth, and total scatter factor, $S_{c}^{*}S_{p}$, where $S_{c}$ is determined from the collimator setting and $S_{p}$ is a function of equivalent square jaw opening. Depth was measured from the patient contour, and shielding was ignored except for the shielding tray transmission factor (TF).

Rectum: Dose was prescribed to isocenter using a three‐field technique, with 6 MV x rays on the posterior and 18 MV on the lateral fields. Wedges were used on the lateral fields in all cases. A single transverse patient contour was manually digitized, and this contour was projected in the superior/inferior direction to form a 3D data set. The contour was also very simple with small (up to 20°) obliquity with respect to the lateral fields. The “hand” calculation was similar to that for prostate with the following additions: irregular equivalent square field calculations were done for fields that had shielding covering greater than one quarter of the field using the Clarkson method. These were used for look‐up of TPR and $S_{p}$. Wedge transmission factors, (WF) as a function of field size and depth, were used for the lateral fields.

Brain: Dose was normalized to the isocenter using 6 MV x rays and a three‐field technique with wedging and shielding. Most of the patient data was acquired using CT The patient contour was convex to the treatment beam in two directions, and significant fractions of the beam could splash out into air. The “hand” calculations were similar to those for the rectum. Blocked equivalent squares were calculated to account for the presence of air and shielding.

Breast: This group was subdivided into two‐field and four‐field techniques. The two‐field group was treated using a tangential parallel opposed pair with a constant source to surface distance setup on a Cobalt^60^ unit or an isocentric setup on a 6 MV linac. The four‐field group was treated using a four‐field, asymmetric matched technique with 6 MV x rays.

Cobalt: Treatments were planned using a half‐blocked beam and wedges at a constant SSD of 80 cm. Dose was prescribed to a point located one third of the distance from the posterior border (central axis) to the skin surface at mid separation. A single transverse patient contour in the treatment plane was manually digitized, and this contour was projected to form a 3D data set. An inverse square correction (ISC) was applied in the “hand” calculations. An off‐axis correction (OAR) was not necessary for Cobalt; however, an off‐axis wedge factor (WOAR) was applied to account for transmission through the wedge to an off‐axis prescription point. For look‐up of TPR and $S_{p}$, an equivalent square was calculated at the prescription point using an in‐house computer program based on the differential tissue air ratio (dTAR) method of Cunningham.^14^ Simply, a Clarkson integral of differential TAR is performed as a function of depth and radius from the prescription point. This corrects for the extremely complex patient contour.

6 MV tangents: Treatments were planned using asymmetric collimators and physical wedges. The isocenter was usually located at mid‐separation of the breast as measured at the base of the treatment fields. The prescription point was positioned one third of the distance from the base of the fields to the skin surface at this level. Patient contours are collected as for Cobalt above. The “hand” calculations were similar to those for Cobalt except that off‐axis factors were included and inverse square corrections were not required.

6 MV 4 field: Treatments were planned using asymmetric collimators and physical wedges. The tangent fields are treated with a nondivergent match to the supraclavicular fields. The isocenter is chosen so that its projection inferiorly to mid‐breast is at mid‐separation at the base of the treatment volume. The prescription point is located one third of the distance from this point to the skin surface at this level. Transverse patient contours are manually digitized at approximately 1 cm intervals. The “hand” calculation method is similar to that used for the 6 MV tangent fields. The supraclavicular prescription point is positioned at mid‐separation, 3 cm superior to the matchline, on a sagittal plane that passes through the center of the treatment field. Sagittal patient contours are manually digitized. The fields are wedged and blocked. The “hand” calculation is similar to that for the rectum and brain with the addition of WOAR and OAR factors to account for off‐axis prescription points. For “hand” calculations, the OAR and WOAR are taken to be invariant with depth.

**Table III:** Statistical summary of MU ratios for breast treatments by technique.

Statistic/Technique	Cobalt	6 MV	Four‐field tangent	Four‐field supraclav
Number of Fields	4113	1906	755	679
Average MU ratio	1.007	1.013	1.017	1.032
St. dev. MU ratio	0.015	0.010	0.018	0.012
Min. MU ratio	0.966	0.954	0.957	0.987
Max. MU ratio	1.072	1.061	1.072	1.077

## RESULTS AND DISCUSSION

**Figure 1:**
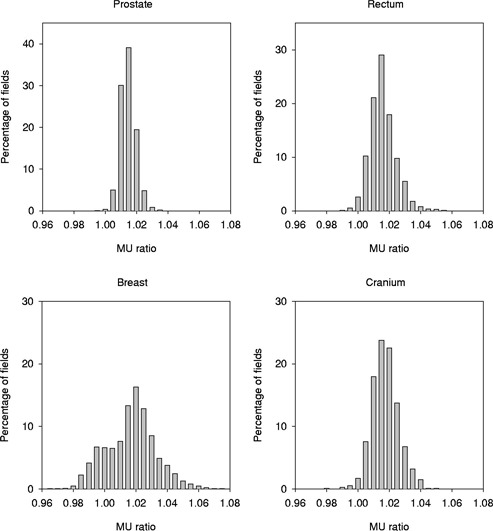
A histogram showing the distribution of MU ratios by site for Pinnacle^3^ TPS compared with “hand” calculations.

The MU ratios presented are derived from over 13‐500 treatment fields over a three‐and‐one‐half‐year period. Summary statistics by site are listed in Table II, and histograms depicting the distribution of these data are given in Fig. 1. For all four treatment sites the average value of the MU ratio is near 1.01; that is, there is an average 1% difference between the treatment planning system and the “hand” monitor unit calculations. Starkschall *et al.* ^6^ reported similar systematic differences of 0.5% to 1.0% using the Pinnacle^3^ planning system and speculated that the discrepancy may be related to differences in the determination of the beam entry point resulting from the voxel size of the calculation grid. For our data, the standard deviation of the MU ratio is smallest for prostate at 0.5% and is greatest for breast at 1.6%, with the rectum and brain intermediate to these. The trends for the minimum and maximum MU ratio with treatment site follow those for the standard deviations.

**Figure 2:**
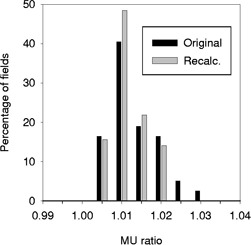
A histogram showing the distribution of MU ratios for Pinnacle^3^ TPS compared with “hand” calculations for prostate. The original “hand” calculation did not account for small corner blocks in the field, whereas the recalculation did.

Looking at each site separately reveals further details. For prostate, the treatment conditions closely simulated the calibration conditions in water phantoms in that mostly square fields were used and incidence surfaces were relatively flat. We suspected that MU differences greater than 2% were due to shielding that was not taken into account by the “hand” calculation. For a subset of 100 treatment fields we calculated equivalent squares for all fields and recalculated the monitor units. These results are shown in Fig. 2. The MU ratio was lowered in all cases and values were well within 2%, although the mean difference was lowered by only 0.1% (data not shown). This retrospective look lead to a change in our current practice and irregular field calculations are done more frequently when the blocking approaches 15% of the field.

For rectal fields, MU differences greater than 2% occurred for large patients where the depths were great and for lateral beams incident on sloping patient contours. This was also true for brain fields where the patient incident surface was convex in two directions. Patient contour was the main factor for any MU ratio that varied by greater than 2% for these fields. To investigate this further, a subset of 30 brain fields were recalculated on the TPS changing the patient contour to a flat surface. The monitor units for the true patient contour were systematically higher than for the flat patient contour by an average value of 0.6%. There were some fields, particularly when the prescription point was asymmetric in the cranium, with differences as high as 2.5%. These differences are consistent with the differences in average and maximum MU ratios between the prostate and brain fields in Table II.

**Figure 3:**
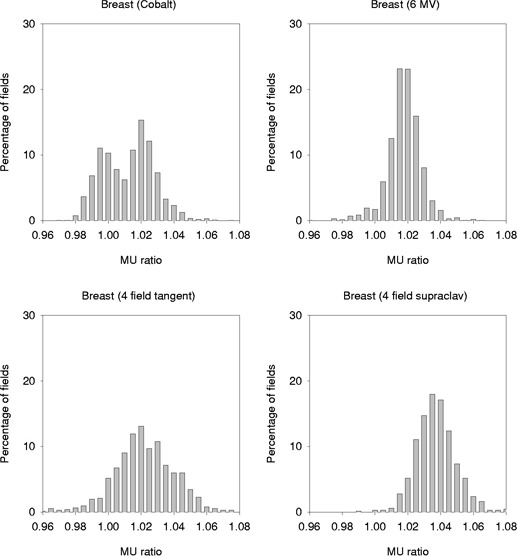
A histogram showing the distribution of MU ratios by treatment technique for Pinnacle^3^ TPS compared with “hand” calculations for breast treatment.

The breast data is shown subdivided by treatment technique. Summary statistics are presented in Table III, and the histograms of the distributions of MU ratio are shown in Fig. 3. The two‐field tangential techniques (Cobalt and 6 MV) have average MU ratios of 1.007 and 1.013, respectively. These are of the same magnitude as the rectal and brain fields. In general, MU ratios were lower for Cobalt^60^ beams than for 6 MV. This may be due to the fact that the Cobalt machine was optimized on the treatment planning system specifically for breast calculations, whereas 6 MV was optimized for general use over a variety of treatment sites. For both energies, patient contour was the main factor for any MU difference greater than 2%. Postmastectomy chestwall contours and breast contours that were not symmetric (steep oblique medial slope and lateral fullness) had larger MU ratios.

The four field breast technique had the highest MU ratios for the entire study. The four‐field tangent fields had an average MU difference 0.5% higher than other 6 MV tangent fields and a standard deviation twice as high as the two‐field counterpart. This is probably due to the fact that the patient surface is extremely complicated in three dimensions, whereas the “hand” calculation assumes the same contour throughout the entire field.

**Table IV:** Mean MU ratios for supraclavicular fields with decreasing levels of complexity.

		Average MU ratio	
Plan Geometry	Component of Complexity	Anterior	Posterior
Original Plan	‐	1.028	1.027
Wedges Removed	WF and WOAR	1.024	1.021
Flat contour, no shielding	dTAR equivalent square	1.016	1.017
X and Y jaws reversed	Collimator exchange effect	1.013	1.014
Asymmetric field size and Prescription point position	OAR	1.011	1.011

**Figure 4:**
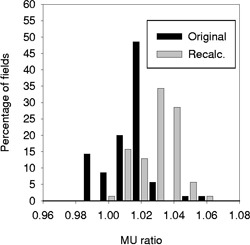
A histogram showing the distribution of MU ratios for Pinnacle^3^ TPS compared with “hand” calculations for breast tangent fields. The original hand calculation used an equivalent square based on patient contour whereas the recalculation assumes a flat patient entrance surface and corrects only for beam “splash” beyond the patient contour.

The methodology we use to calculate the dTAR equivalent square for two‐ and four‐field tangent breast fields is perhaps unique to our center. An alternative approach is to calculate an equivalent square assuming a constant tissue depth with an open field that matches the amount of breast in the beam, i.e., correcting for splashes into the air. This is determined from the projection of the breast on a simulator film or digitally reconstructed radiograph (DRR). In order to compare these two approaches we re‐calculated the monitor units for a subset of breast patients (20 each for Cobalt and 6 MV and 10 for 6 MV four field) using both equivalent squares. The results are shown in Fig. 4. The average MU ratio is higher by 1.5% using the simpler equivalent square, and this difference is consistent for all three treatment techniques (data not shown). This supports our conclusion that much of the difference is due to patient contour.

The supraclavicular fields have an average MU difference slightly greater than 3%. In order to identify the sources of these differences a more detailed analysis was performed on a subset of 17 patients. These patients were replanned using progressive simplifications in planning geometry and the mean MU ratios were evaluated. The results are presented in Table IV. As the level of complexity of the plan was decreased, the mean MU difference decreased until it matched those for the simple prostate plans. The source of the higher MU ratios is a cumulative effect due to differences in a number of factors in the monitor unit calculation. This effect is also manifest in a higher standard deviation in MU ratios for supraclavicular fields compared with prostate fields.

## CONCLUSIONS

This report has presented a detailed analysis of differences between the monitor unit calculations of the Pinnacle^3^ treatment planning system and a simple “hand” calculation for four treatment sites of varying complexity. We have identified systematic differences in the calculation methods and determined the range of random dose variations for these sites. No errors have been discovered in our investigation of systematic differences. Systematic differences have been attributed to the effects of complex treatment geometry and the limitations of the calculation methods. Where multiple factors are involved in the calculation of monitor units these differences can accumulate to produce somewhat greater net differences.

Analysis of these data have been useful in establishing action thresholds for the investigation of individual treatment plans when “hand” calculations differ from those of the planning system. The action threshold will depend upon the treatment site and the complexity of the planning geometry. Failure to account for individual factors in the “hand” calculation must be accounted for by increasing the action threshold on a site by site basis.
